# Formation Control of Automated Guided Vehicles in the Presence of Packet Loss

**DOI:** 10.3390/s22093552

**Published:** 2022-05-07

**Authors:** Leila Sedghi, Jobish John, Md Noor-A-Rahim, Dirk Pesch

**Affiliations:** School of Computer Science and Information Technology, University College Cork, T12 K8AF Cork, Ireland; j.john@cs.ucc.ie (J.J.); m.rahim@cs.ucc.ie (M.N.-A.-R.); dirk.pesch@ucc.ie (D.P.)

**Keywords:** formation control, packet loss, long short-term memory

## Abstract

This paper presents the formation tracking problem for non-holonomic automated guided vehicles. Specifically, we focus on a decentralized leader–follower approach using linear quadratic regulator control. We study the impact of communication packet loss—containing the position of the leader—on the performance of the presented formation control scheme. The simulation results indicate that packet loss degrades the formation control performance. In order to improve the control performance under packet loss, we propose the use of a long short-term memory neural network to predict the position of the leader by the followers in the event of packet loss. The proposed scheme is compared with two other prediction methods, namely, memory consensus protocol and gated recurrent unit. The simulation results demonstrate the efficiency of the long short-term memory in packet loss compensation in comparison with memory consensus protocol and gated recurrent unit.

## 1. Introduction

Automated guided vehicles (AGVs) along with their formation control are a key technology for Industry 4.0 as they automate the coordinated movement of materials and components in manufacturing environments in a safe, secure, and operationally efficient manner [1,2]. In many applications, formation control refers to the process by which a group of autonomous vehicles follows a predefined trajectory while maintaining a desired spatial pattern [3]. Multi-agent formation control systems have received considerable attention in the research literature due to the inherent difficulties associated with the control and coordination strategies, especially in the absence of a central controller. AGV formation control has been extensively used in smart factories and warehouse environments in a mobile robot-based production line system [4]. Formation control has a wide range of applications, including vehicle platoon control [5], cooperative transportation of large or heavy loads carried by multiple mobile robots, or automated guided vehicles (AGVs) [6]. Formation control [7] addresses various sub-problems such as localization [8], obstacle avoidance [9], and distributed path planning [10], with numerous studies on these topics. Due to its simplicity and scalability, the leader–follower approach is a widely adopted formation control method [11]. This method selects one or more robots as leaders to guide and move along the desired trajectory, while the remaining robots are selected as followers to track the leader(s) paths. A leader–follower formation control problem can be considered a tracking problem in control systems, where the leader moves along the desired trajectory, and the followers track the leader, maintaining the required formation [3]. We consider that each robot’s (e.g., leader, follower) control procedure only uses local measurements and is not based on a centralized controller. Consensus control is another type of formation control in which all robots coordinate and make decisions based on information from their neighbours to achieve consensus [12].

Generally, the AGVs/robots exchange formation control information through a wireless network [10], which plays a vital role in interconnecting the AGVs. Formation control is vulnerable to wireless network uncertainties such as wireless channel noise, communication delay, and communication packet losses. These uncertainties can result in a variety of complex dynamic behaviours in control systems, such as divergence, oscillation, and instability [13]. Most existing studies assume ideal communication links and do not consider the effect of packet loss or delay [14,15,16]. There are only a few works that consider communication delay, while the packet loss is generally ignored [12,17,18,19,20,21].

However, packet loss in the communication network has a significant impact on the performance of any industrial process control system [22], specifically the trajectory tracking performance of a robotic formation. In the formation control of robots, holonomic and non-holonomic robots are two types of robots which are widely used in industry; while holonomic robots move regardless of their heading angle/orientation, non-holonomic robots such as differential drive robots are unable to move into arbitrary directions due to a non-holonomic constraint. As a result, they require sophisticated control mechanisms, and the impact of packet loss on the formation control of non-holonomic differential drive robots is significant. Holonomic robot formation is more resilient to network uncertainties than non-holonomic robot formation [23]. To mitigate the effects of packet loss, communication networks typically use re-transmissions to ensure that the data are received at the intended receiver. However, re-transmission will increase the transmission delay [18]. On the other hand, a range of control approaches such as robust control [24], model predictive control [25], sliding mode control [13], and various estimators [26] or filters [27] are widely used to address packet loss from a control perspective.

Machine learning can also be a useful tool to mitigate packet loss, and its efficiency has been demonstrated in a variety of wireless network applications within the Internet of Things context, including network management, congestion avoidance, and resource allocation optimization [28]. As a result, various studies have been conducted on the predictive capabilities of machine learning, particularly deep learning approaches such as long short-term memory (LSTM) [29]. To the best of our knowledge, no prior research has used deep learning techniques to address the effect of packet loss on formation control. Inspired by the above discussions, we propose using LSTM to control the formation of non-holonomic differential drive AGVs in the presence of packet loss. LSTM is a powerful technique for forecasting time series and classifying data [30]. The LSTM algorithm is capable of extracting features from time series over a longer time span and of solving the vanishing gradient problem [31]. In this study, the leader follows a trajectory, which is a sequence of locations over time. Thus, LSTM is proposed to forecast the leader’s position, and followers use this predicted value as needed (whenever the position data sent by the leader is lost by the wireless network). As the quality of the wireless communication deteriorates, followers frequently predict the leader’s position using LSTM for tracking the leader’s trajectory. Our results show that LSTM outperforms gated recurrent units (GRUs) and memory consensus protocol (MCP). MCP is a well-known technique that stores each neighbour’s previously received data and uses them when a packet is not received by a neighbour [32]. MCP becomes inefficient as the number of consecutive packet losses increases [33]. The GRU architecture is similar to the LSTM, but it lacks an output gate, which reduces the calculation burden and simplifies implementation [34]. However, LSTM prediction usually outperforms GRU prediction [35]. We model the packet loss using a Bernoulli distribution and evaluate LSTM predictions with a packet loss of 30 and 50 percent.

The following are the major contributions of this paper:

- Study of decentralized leader–follower formation control for non-holonomic automated guided vehicles using linear quadratic regulator (LQR). LQR is a simple yet popular control approach that can be easily implemented and that has not yet been used for formation control.
- Analysis of the impact of packet loss on the formation control of AGVs.
- Improving the performance of a linear quadratic regulator (LQR) controller via machine learning, e.g., LSTM, to deal with packet losses, rather than using a highly non-linear and complicated controller such as a sliding mode controller.
- Development of a mechanism to compensate for packet loss with LSTM and the application of the mechanism to the formation control of non-holonomic differential drive robots, which are more sensitive to network uncertainties due to non-holonomic constraints.
- Comparing LSTM with GRU and MCP for the compensation of 30 and 50 percent packet loss through simulation in MATLAB/SIMULINK.

The rest of this paper is organized as follows. In Section 2, related work focused on the compensation of packet loss for AGVs is presented. Section 3 discusses how to model differential drive robots mathematically. A controller is designed in Section 4. In Section 5.1, the vulnerability of the system to packet loss is demonstrated. In Section 5.2, an LSTM network is used to counteract the negative impact of packet loss. Prediction accuracy of LSTM, GRU, and MCP are compared in Section 6.1. Simulations are used to evaluate the proposed method’s performance in Section 6.2. Finally, the conclusion and suggestions for future work are presented in Section 7.

## 2. Related Work

In this section, we mainly discuss prior research that has developed algorithms and controllers to deal with packet loss in the formation control of AGVs. The authors in [36] concluded that by reducing the number of control updates and transmissions, the wireless communication channel is less congested and hence packet losses can be minimized. However, the authors did not propose any mechanisms to address the effect of packet loss, which can affect the performance of their formation control.

In [33], a networked predictive controller and two algorithms are developed to cope with consecutive packet loss and communication delay for non-linear wheeled mobile robots. The authors simulated the effect of packet loss by considering a tunnel the robot travels through that is no longer detectable with cameras and associated servers [33]. A well-quantified analysis of packet loss and its effects is missing.

A consensus-based tracking control strategy was studied for leader–follower formation control of multiple mobile robots under packet loss [37]. A novel multiple Lyapunov functional candidate and linear matrix inequality (LMIs) ensure that the robots reach consensus when packet loss and communication weight (representing the rate of information flow between agents) are taken into account. Packet loss is modelled using a Bernoulli distribution and assumed to be 20% in the majority of scenarios. It is shown that the system can achieve consensus under high packet loss, but it also takes a long time to reach consensus. Their simulation results consider that one of the agents has access to the maximum amount of information, implying that their communication weight is equal to one.

In [13], event-triggered second-order sliding mode control is designed for consensus-based formation control of non-holonomic robots. Although sliding mode control is a robust method for counteracting packet loss and delay, the second-order sliding mode controller is a difficult controller to implement in real-world industrial scenarios. Furthermore, the main focus of this article is on event-triggered control, and the highest packet loss rate considered is 20%, modelled using the Bernoulli distribution for a circular trajectory.

Among the learning methods used to address packet loss, iterative learning control (ILC) design has been used to cope with packet loss in several articles [38,39]. ILC is based on the concept of learning from previous iterations in order to improve the performance of a system that repeatedly performs the same task [40]. In [41], ILC is applied to a linear system that suffers from 15% and 25% packet loss. The authors show that after 50 iterations, the system compensates for this packet loss. ILC is also used in non-linear multi-agent systems to solve the consensus problem of a leader–follower use case with packet dropouts of 10% and 40% [42]. None of these ILC studies [38,39,41,42] were conducted with non-holonomic AGV formation control, which is a more challenging system because of the non-holonomic constraints.

It is worth noting the difference between the methods proposed in this research and the dead reckoning method. They might seem similar in definition but they have totally different approaches and functions. Dead reckoning (the “deduced reckoning” of sailing days) is a simple and basic mathematical procedure for finding the present location of a vessel by advancing some previous position through a known course and using the velocity information of a given length of time [43]. In dead reckoning, the Global Positioning System (GPS) is not available, e.g., no GPS receiver, indoor environment, etc. [44]; therefore, dead reckoning is used as a localization method that estimates the robot’s position, orientation, and integrates local sensor information over time, which usually suffer from drifts [45]. In this research, we do not address localization, and it is considered that each robot knows its own position accurately (e.g., the global reference is available).

In contrast to previous research, this study focuses on the use of deep learning to compensate for packet loss while robots maintain their formation. When packet loss occurs, LSTM, GRUs, and MCP are optional methods for predicting the leader position. LSTM is used for AGVs in various fields such as path planning [46], state estimation and sensor fusion of holonomic robots [47], data fusion of the odometry and IMU [48] anomaly detection [49] and fault detection [50]; however, no research has been conducted to compensate for packet loss in the formation control of AGVs via deep learning.

## 3. Mathematical System Model

**Figure 1:**
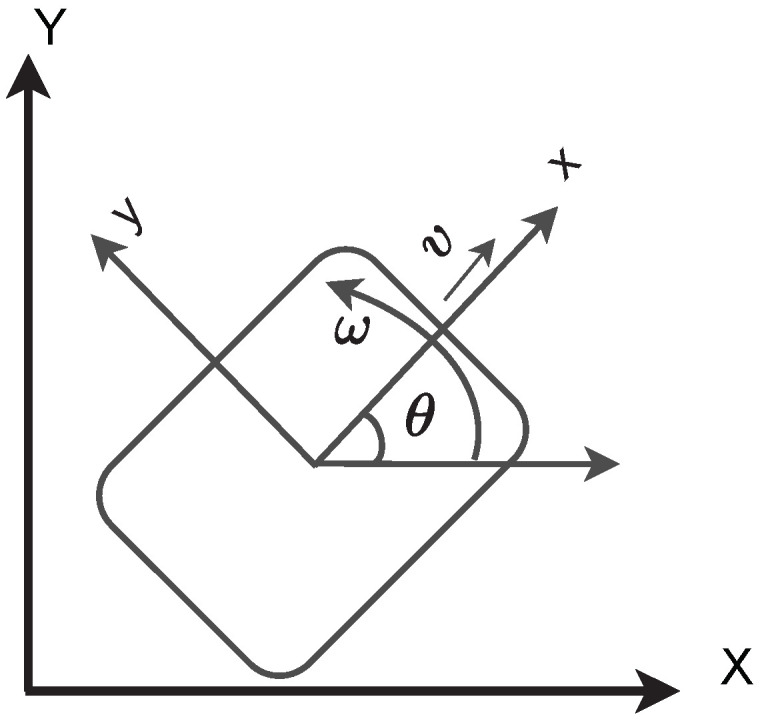
Kinematic model of non-holonomic robot.

In this section, we consider the generic mathematical system model of non-holonomic robots moving in the X–Y plane [51]. We chose a non-holonomic differential drive robot, which is widely used for AGVs in industry, as detailed in Section 1. In non-holonomic robots, the number of control variables is less than the number of state variables, which complicates formation control. This section discusses non-holonomic constraints to derive a kinematic model of non-holonomic robots. The differential robot’s kinematics can be simplified using unicycle model equations [51] in which the wheel is assumed to have a desired velocity at a specified heading angle. As shown in Figure 1, the robot’s position is determined by the co-ordinate (*x*, *y*, θ), which is the robot’s orientation relative to the axes (X and Y). There are also two control inputs denoted by *v* and ω, which correspond to linear and angular velocity, respectively.

(1) $$\begin{array}{cc} \dot{x} & =vcos\theta \\ \dot{y} & =vsin\theta \\ \dot{\theta } & =\omega \end{array}$$

The reference trajectory followed by the leader is represented by Equation ($x_{ref},\;y_{ref}$, $\theta_{ref}$), where $\theta_{ref}$ is the reference heading angle (tangent angle of each point on the path), which can be obtained from the reference positions ($x_{ref},\;y_{ref}$) given by Equation (2).

(2) $$\theta_{ref}=\arctan\frac{{\dot{y}}_{ref}}{{\dot{x}}_{ref}}+k\pi ,k\epsilon \left(0,1\right)$$

k=0 represents the forward drive direction and k=1 represents the reverse drive directions.

The linear velocity $v_{ref}$ of the robot is obtained with Equation (3) and the reference angular velocity $\omega_{ref}$ is obtained with Equation (4).

(3) $$v_{ref}=\pm \sqrt{{\dot{x}}_{ref}^{2}+{\dot{y}}_{ref}^{2}}$$

where the sign relates to the desired drive direction (+ for the forward direction and − for the reverse direction)

(4) $$\omega_{ref}=\frac{{\dot{x}}_{ref}.{\ddot{y}}_{ref}-{\dot{y}}_{ref}.{\ddot{x}}_{ref}}{{\left({\dot{x}}_{ref}\right)}^{2}+\left({\dot{y}}_{ref}^{2}\right)}$$

## 4. Controller Design

**Figure 2:**
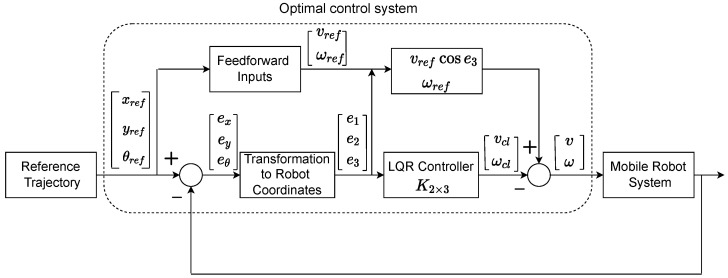
Mobile robot controller.

In this section, we detail the design of the linear quadratic regulator (LQR) tracking controller used for controlling the non-holonomic robots, so that they follow a desired trajectory through a leader–follower strategy. A similar design is detailed in [52] for a single robot following a reference trajectory. LQR is an optimal control technique which considers the states of the system and control inputs when making optimal control decisions and computes the state feedback control gain [53]. LQR was chosen because of its simplicity and ease of implementation, while providing good accuracy, as shown in [52] for a single AGV. The designed LQR is a simple controller in comparison with non-linear controllers such as a sliding controller, which has been widely used for AGVs in recent years. As shown in Figure 2, the kinematic controller (LQR controller) generates two control signals, the angular velocity ($\omega_{cl}$) and the linear velocity ($v_{cl}$), for each robot’s trajectory tracking. The current position of the robot (*x*, *y*, and θ) is compared with its expected reference trajectory ($x_{ref}$,$y_{ref}$ and $\theta_{ref}$) and the trajectory tracking errors are fed to the LQR controller after the required transformations [52].

To design the LQR controller, let us consider a linear time-invariant (LTI) system:

(5) $$\begin{array}{cccc} \dot{x} & =Ax(t)+Bu(t), & t\geq 0, & x(0)=0\\ \dot{y} & =Cx(t)+Du(t), & t\geq 0 \end{array}$$

where $A\in R^{n\times n}$, $B\in R^{n\times m}$, $C\in R^{p\times n}$, and $D\in R^{p\times m}$ are the system matrix, control matrix, output matrix, and feed forward matrix, respectively, representing the state space model. The dimensions of these matrices are depicted with *n* state variables, *m* inputs, and *p* outputs. *x* is the state vector, *u* is the control vector, and *y* is the output vector. The LQR controller generates the control input that minimizes the cost function [54] given by Equation (6).

(6) $$J\left(u\right)=\int_{0}^{\infty }\left[\;x^{T}\left(t\right)Qx\left(t\right)+u^{T}\left(t\right)Ru\left(t\right)\right]dt$$

where $Q=Q^{T}$ is a positive semi-definite matrix that penalizes the departure of system states from the equilibrium, and $R=R^{T}$ is a positive definite matrix that penalizes the control input [55]. The feedback control law that minimizes the value of the cost function is given by Equation (7):

(7) u=−Kx

where *K*, the optimal state feedback control gain matrix, is obtained with Equation (8):

(8) $$K=R^{-1}B^{T}P$$

and *P* is found by solving the algebraic Riccati Equation (ARE) (9) [56]:

(9) $$A^{T}P+PA+Q-PBR^{-1}B^{T}P=0$$

Thus, to design an LQR controller, the trajectory tracking problem should be written in the form of Equation (5). This gives the trajectory tracking errors in the form of Equation (10).

(10) $$\left[\begin{array}{c} e_{x}\\ e_{y}\\ e_{\theta } \end{array}\right]=\left[\begin{array}{c} x_{ref}-x\\ y_{ref}-y\\ \theta_{ref}-theta \end{array}\right]$$

where $e_{x}$, $e_{y}$, and $e_{\theta }$ are errors in *x*, *y*, and heading angle, respectively. To transform these errors into robot coordinates, a rotation matrix was applied to the system as stated in Equation (11).

(11) $$\left[\begin{array}{c} e_{1}\\ e_{2}\\ e_{3} \end{array}\right]=\left[\begin{array}{ccc} \cos\theta & \sin\theta & 0\\ -\sin\theta & \cos\theta & 0\\ 0 & 0 & 1 \end{array}\right].\left[\begin{array}{c} e_{x}\\ e_{y}\\ e_{\theta } \end{array}\right]$$

The transformed errors ($e_{1}$, $e_{2}$, $e_{3}$) are fed to the LQR controller and it generates the required control signals $\omega_{cl}$ and $v_{cl}$, as shown in Figure 2.

Applying Equation (1) to the time derivative of Equation (11) yields the state space model given by Equation (12).

(12) $$\left[\begin{array}{c} \dot{e_{1}}\\ \dot{e_{2}}\\ \dot{e_{3}} \end{array}\right]=\left[\begin{array}{cc} \cos e_{3} & 0\\ \sin e_{3} & 0\\ 0 & 1 \end{array}\right].\left[\begin{array}{c} v_{ref}\\ \omega_{ref} \end{array}\right]+\left[\begin{array}{cc} -1 & e_{2}\\ 0 & -e_{1}\\ 0 & -1 \end{array}\right].\left[\begin{array}{c} v\\ \omega \end{array}\right]$$

where

(13) $$\begin{array}{cc} vs. & =v_{ref}\cos e_{3}-v_{cl}\\ \omega & =\omega_{ref}-\omega_{cl} \end{array}$$

Applying Equation (13) to Equation (12) results in

(14) $$\left[\begin{array}{c} \dot{e_{1}}\\ \dot{e_{2}}\\ \dot{e_{3}} \end{array}\right]=\left[\begin{array}{ccc} 0 & \omega & 0\\ -\omega & 0 & 0\\ 0 & 0 & 0 \end{array}\right].\left[\begin{array}{c} e_{1}\\ e_{2}\\ e_{3} \end{array}\right]+\left[\begin{array}{c} 0\\ \sin e_{3}\\ 0 \end{array}\right].v_{ref}+\left[\begin{array}{cc} 1 & 0\\ 0 & 0\\ 0 & 1 \end{array}\right].\left[\begin{array}{c} v_{cl}\\ \omega_{cl} \end{array}\right]$$

Linearizing Equation (14) around the operating point ($e_{1}=e_{2}=e_{3}=0,v_{cl}=\omega_{cl}=0$) [51], the state space model of the linear system given by Equation (15) is obtained.

(15) $$\left[\begin{array}{c} \dot{e_{1}}\\ \dot{e_{2}}\\ \dot{e_{3}} \end{array}\right]=\left[\begin{array}{ccc} 0 & \omega_{ref} & 0\\ -\omega_{ref} & 0 & v_{ref}\\ 0 & 0 & 0 \end{array}\right].\left[\begin{array}{c} e_{1}\\ e_{2}\\ e_{3} \end{array}\right]+\left[\begin{array}{cc} 1 & 0\\ 0 & 0\\ 0 & 1 \end{array}\right].\left[\begin{array}{c} v_{cl}\\ \omega_{cl} \end{array}\right]$$

Comparing Equation (15) with the standard form in Equation (5), the system is controllable if and only if its controllability matrix (R = [$B,AB,A^{2}B$]) has a full rank. However, rank (R) = 3 if either $v_{ref}$ or $\omega_{ref}$ are non-zero, which is a sufficient condition only when the reference inputs $v_{ref}$ and $\omega_{ref}$ are constant. This happens only when the trajectory is a line or a circular path. The controllability of a driftless system can be derived from Chow’s theorem if the system is completely non-holonomic [51]. The robot model represented by Equation (1) is completely non-holonomic since it has only one non-holonomic constraint, which is represented by Equation (16):

(16) $$\dot{x}\sin\theta -\dot{y}\cos\theta =0$$

Therefore, the robot cannot move in a lateral direction due to its wheels and it is controllable [51], as shown in Figure 2, where the LQR controller is given by Equation (17) and $K_{2\times 3}$ is the gain matrix with three states and two inputs.

(17) $$\left[\begin{array}{c} v_{cl}\\ \omega_{cl} \end{array}\right]=-\left[\begin{array}{ccc} k_{11} & k_{12} & k_{13}\\ k_{21} & k_{22} & k_{23} \end{array}\right].\left[\begin{array}{c} e_{1}\\ e_{2}\\ e_{3} \end{array}\right]$$

To obtain the LQR controller gain ($K_{2\times 3}$), matrices Q and R are tuned, where *Q* is a positive-definite/semi-definite diagonal matrix related to the state variables, and *R* is a positive-definite diagonal matrix related to the input variables [57]. The following *Q* and *R* were selected according to [52] for the evaluation of our tracking system.

$$Q=\left[\begin{array}{ccc} 1 & 0 & 0\\ 0 & 1 & 0\\ 0 & 0 & 10 \end{array}\right],R=0.0001\left[\begin{array}{cc} 1 & 0\\ 0 & 1 \end{array}\right]$$

## 5. Formation Control under Packet Loss

In this section, we first evaluate the controller’s performance under various packet loss conditions. Following that, we discuss the application of an LSTM model to a follower in order to predict the position of the leader when packet loss occurs.

### 5.1. Impact of Packet Loss on Formation Control

**Figure 3:**
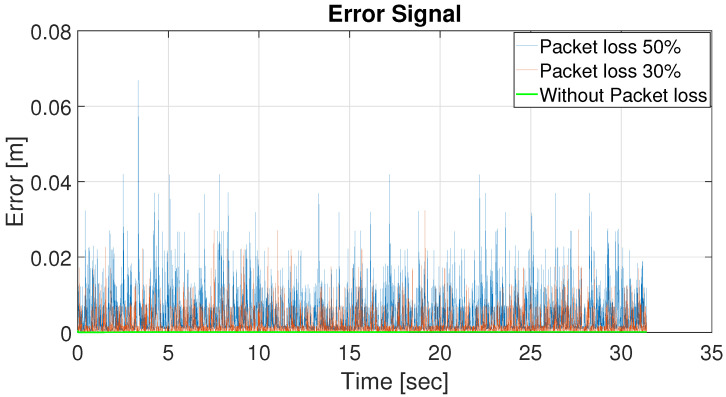
Effect of packet loss.

As demonstrated in [52], the LQR controller performs admirably in tracking the trajectory of a single robot along a variety of paths. Here, we extend the LQR tracking control problem [52] to the formation control of multiple robots in various packet loss scenarios. In the leader–follower approach, the leader’s position is communicated to all followers at regular intervals. The communication interval is considered to be 0.05 s and the sampling interval is 0.005 s. The effects of packet loss are depicted in Figure 3, which illustrates how packet loss results in an increased follower position error of around 4 cm. The simulation results in Figure 3 were obtained by considering a memory element in each follower robot that stores the most recent position of the leader. That is, whenever packet loss occurs, a follower makes use of the last received data stored in its memory to track the reference (leader) trajectory. This approach is called MCP, as detailed in Section 2. Here, we apply an LSTM prediction model to alleviate the impact of packet loss and we compare the system’s performance with MCP and GRU.

### 5.2. Long Short-Term Memory to Cope with Packet Loss

Our objective is to enhance the optimal control system (as shown in Figure 2) using LSTM rather than designing a highly non-linear tracking controller. We believe that, with the recent advancements in machine learning techniques, the existing industrial controller’s performance can be improved in the presence of various network uncertainties such as packet loss, delays, etc. We propose to use LSTM, a type of recurrent network that reuses previously stored data and its dependencies, for predicting the latest position of the reference trajectory (leader). LSTM has been widely used for the prediction of time series data [58,59,60]. As we are attempting to predict the leader’s trajectory, which is a time-based ordered sequence of locations, this problem fits within the LSTM framework. LSTM has been addressed in numerous articles [61,62,63] in order to learn and remember long-term dependency and information. By incorporating various gates, such as an input gate, an output gate, and a forget gate, LSTM is expected to improve traditional recurrent neural networks (RNNs). These various gates enable LSTM to achieve a trade-off between the current and the previous inputs while alleviating an RNN’s vanishing gradient and exploding gradient problems [61]. We detail the LSTM model along with various gates/parameters and evaluate the system’s performance in the following sections.

#### 5.2.1. Architecture of LSTM Prediction and Control

A basic LSTM network for prediction begins with an input layer, followed by an LSTM layer, a fully connected layer, and finally, a regression output layer. The input layer provides the position of the leader to the LSTM layer. The hidden layer is in charge of storing and remembering the position data received from the leader. The output layer provides the leader robot’s predicted position. Since the position of the leader is characterized by its x and y position and heading angle, we use three independent LSTM neural network models for predicting each of these states.

**Figure 4:**
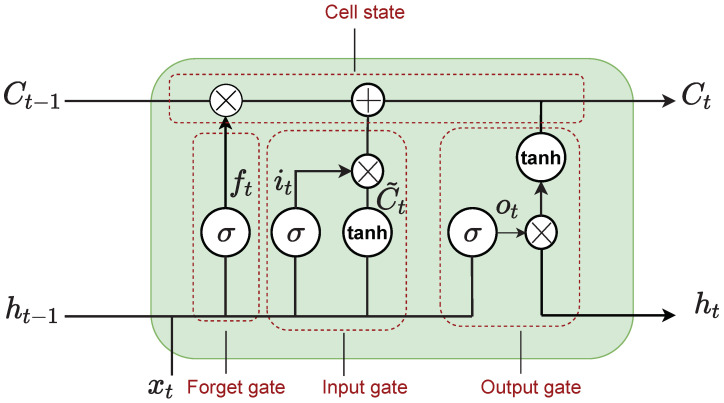
LSTM architecture.

As illustrated in Figure 4, LSTM is equipped with a “gate” structure that enables it to add or remove cell state information and selectively pass the information while passing through different gates as detailed below [61]:

- Forget gate: The forget gate ($f_{t}$), given by Equation (18), decides whether the information from the previous cell state $C_{t-1}$ should be discarded or not.
  (18) $$f_{t}=\sigma \left(W_{f}.\left[h_{t-1},x_{t}\right]+b_{f}\right)$$
  where $f_{t}$ is the forget gate, σ is the sigmoid function, $W_{f}$ is the weight matrix, $b_{f}$ is the bias term, $h_{t-1}$ is the previous hidden layer output, and $x_{t}$ is the new input.
- Input gate: This gate determines the information that has to be stored in the cell states that includes two parts given by Equation (19). The first part in Equation (19) consisting of σ identifies which value is to be updated, and the second part in Equation (19) including tanh generates the new candidate values.
  (19) $$\begin{array}{cc} i_{t} & =\sigma (W_{i}.\left[h_{t-1},x_{t}\right]+b_{i})\\ \tilde{C_{t}} & =\tanh\left(W_{C}.\left[h_{t-1},x_{t}\right]+b_{C}\right) \end{array}$$
  where $i_{t}$ is the input gate, $\tilde{C_{t}}$ is the candidate state of the input, and σ and tanh are the sigmoid and hyperbolic tangent functions, respectively. $W_{i}$ and $W_{C}$ are the weight matrices, $b_{i}$ and $b_{C}$ are the bias terms, $h_{t-1}$ is the previous hidden layer output, and $x_{t}$ is the new input.
- Updating cell state: Updating the cell state considers the new candidate memory and the long-term memory given by Equation (20).
  (20) $$C_{t}=f_{t}\times C_{t-1}+i_{t}\times \tilde{C_{t}}$$
  where $C_{t}$ and $C_{t-1}$ are the current and previous memory states, $f_{t}$ is the forget gate, $i_{t}$ is the input gate, and $\tilde{C_{t}}$ is the input candidate state.
- Output gate: This gate determines the output of the LSTM given by Equation (21).
  (21) $$\begin{array}{cc} o_{t} & =\sigma (W_{o}.\left[h_{t-1},x_{t}\right]+b_{o})\\ h_{t} & =o_{t}\times \tanh C_{t} \end{array}$$
  where $o_{t}$ is the output gate. $W_{o}$ and $b_{o}$ are the weight matrix and bias terms, respectively. $h_{t-1}$ and $h_{t}$ are the previous and current hidden layer outputs, $x_{t}$ is the new input, and $C_{t}$ is the current state of the memory block. The first part in Equation (21), which includes σ, determines which part of the cell state will be output ($o_{t}$), and the second part in Equation (21) processes the cell state by tanh multiplied by the output of the sigmoid layer.

#### 5.2.2. Application of LSTM for Leader Position Prediction

As previously stated, follower robots should have access to the latest position of the leader in order to maintain accurate formation control. When packet loss occurs, followers are unaware of the leader’s true position. To cope with this, we use an LSTM for predicting the leader’s trajectory. The LSTM network is trained using the leader trajectory and then its states are updated.

**Figure 5:**
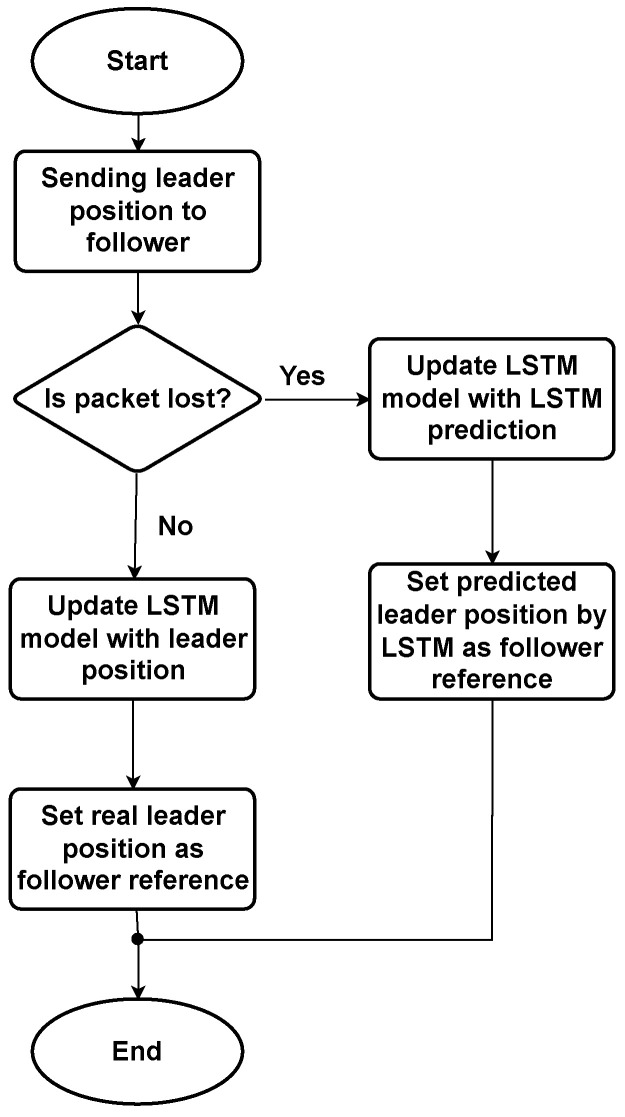
The flowchart of the proposed approach of sending the leader position to the follower.

As shown in Figure 5, when no packets are lost, network states are updated with the actual observed leader position. In the event of packet loss, network states are updated using previous LSTM predictions, as observed leader position values are unavailable.

## 6. Performance Evaluation

In this section, we evaluate the performance of the different prediction schemes LSTM, GRU, and MCP. We also evaluate the performance of the leader–follower formation control system with these prediction methods in different packet loss scenarios. All the performance evaluations are carried out through MATLAB/SIMULINK simulations.

### 6.1. Prediction Accuracy of LSTM, GRU, and MCP

**Figure 6:**
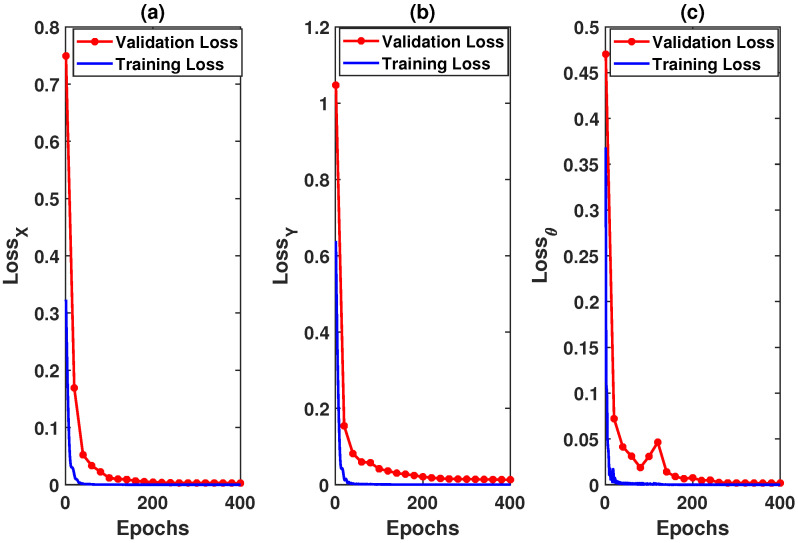
Training and validation loss for LSTM: (**a**) loss for X; (**b**) loss for Y; (**c**) loss for heading angle.

Here, we discuss the prediction performance of LSTM, GRU, and MCP for a circular trajectory for 30% and 50% packet loss. LSTM was trained with the leader’s trajectory positions; 80% of these data were used for training the LSTM and 20% was used for validation. Figure 6 shows the validation and training loss for LSTM. Over the 400 time periods, the proposed LSTM model was able to learn to predict with the desired accuracy.

**Figure 7:**
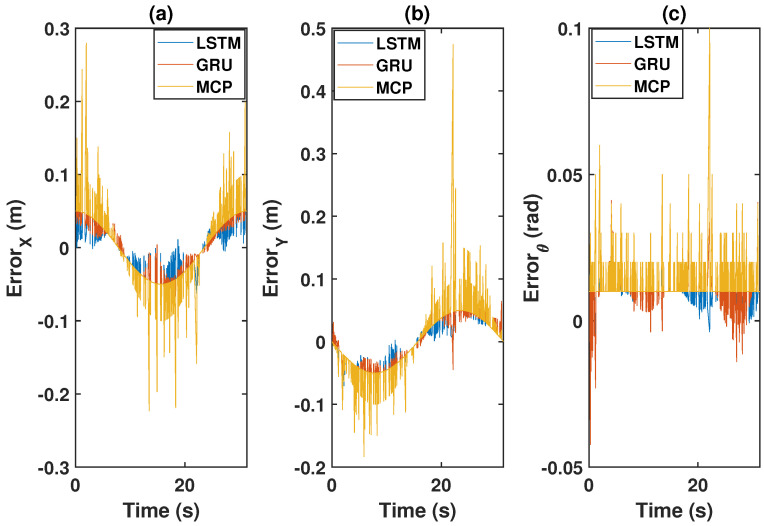
Prediction errors: (**a**) error for X; (**b**) error for Y; (**c**) error for heading angle.

The errors between the actual and the predicted positions of the leader trajectory (X, Y, and heading angle) are shown in Figure 7. As shown in the figure, LSTM provided more accurate predictions than GRU and MCP. The root mean square error (RMSE) between the actual and predicted positions is shown in Table 1. From Table 1, it is clear that LSTM provided the most accurate prediction in comparison with GRU and MCP for both 30% and 50% packet loss scenarios. MCP had the worst prediction performance.

### 6.2. Simulation Results

Here, we discuss the formation control performance of four robots with one of them acting as leader. For each robot, the controller diagram depicted in Figure 2 was simulated using MATLAB/SIMULINK. The followers and the leader attempted to maintain their formation as they travelled along a pre-defined path. At regular communication intervals, the leader’s position was communicated to all the followers via wireless broadcast communication. In the event of packet loss, LSTM predicted the leader’s position for the followers. The LSTM and GRU model settings were identical and they are listed in Table 2. The LSTM and GRU model parameters were carefully chosen to maintain a balance among prediction accuracy, computing resources, and calculation time.

In our use case, the follower robot was expected to maintain a predefined distance from the leader. The accuracy of the LSTM location prediction was measured using the RMSE given by Equation (22). RMSE is a frequently used measure of the difference between the predicted and the actually observed values.

(22) $$RMSE=\sqrt{\frac{1}{N}\sum_{i=1}^{N}(x_{i}-{\hat{x}}_{i}{)}^{2}}$$

where $x_{i}$ is the observed value, ${\hat{x}}_{i}$ is the predicted value, and N is the number of data points.

The simulations were carried out with a sampling time of 5 ms and a communication interval of 50 ms. The leader positions communicated to the followers were vulnerable to packet loss, which was modelled using a Bernoulli distribution with a probability of ρ equal to 0.3 and 0.5. Packet loss had a more significant impact on the formation control when the trajectory followed was not simple in nature (e.g., straight line or its variants). We chose circular and eight-shaped paths for our evaluations.

#### 6.2.1. Circular Path

**Figure 8:**
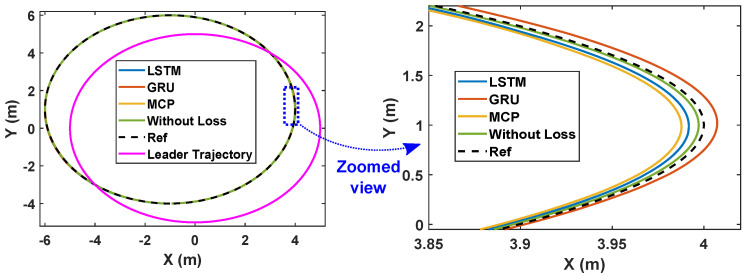
Trajectory of the robots during formation control (packet loss—30%).

**Figure 9:**
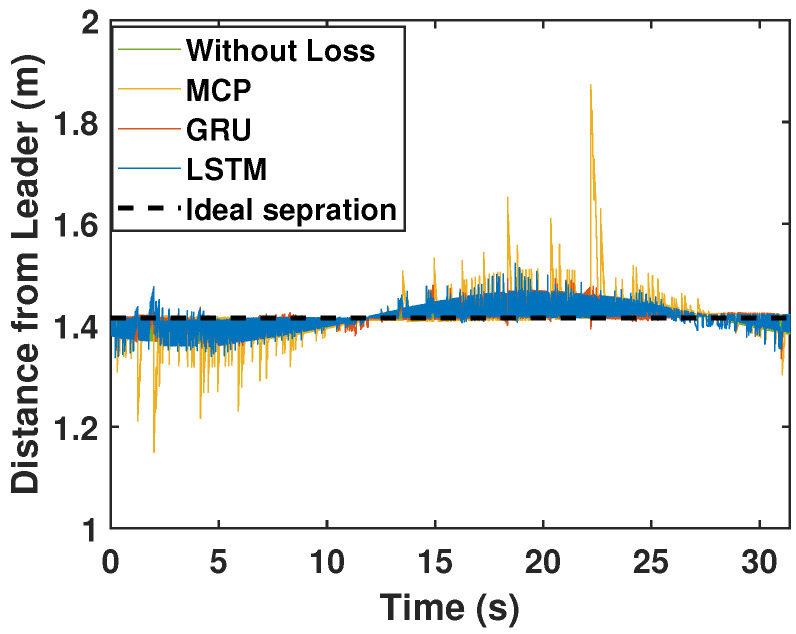
Distance of the follower from the leader for the circular trajectory (packet loss—30%).

Here, we consider the leader and the followers as moving through a circle while packet loss is considered to be 30%. As illustrated in Figure 8, LSTM prediction compensated for packet loss better than GRU and MCP. The distance from the leader is also depicted in Figure 9, which compares the prediction performance of MCP to those of LSTM and GRU. The RMSE values of X, Y, and the heading angle of the follower are shown in Table 3. From Figure 8, it is clear that LSTM-based prediction can provide formation control performance that is comparable to that in perfect communication scenarios (0% packet loss), even with 30% packet loss. This is clearly observed in the RMSE as well. A lower RMSE for X and a close enough RMSE for Y and heading angle can be observed in Table 3 when comparing 0% and 30% packet loss scenarios. This demonstrates how well LSTM prediction can compensate for packet loss. Overall, LSTM performed 10% better than GRU and 148.07% better than MCP.

**Figure 10:**
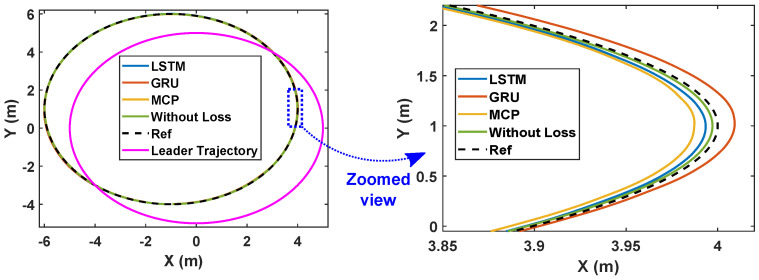
Trajectory of the robots during formation control (packet loss—50%).

**Figure 11:**
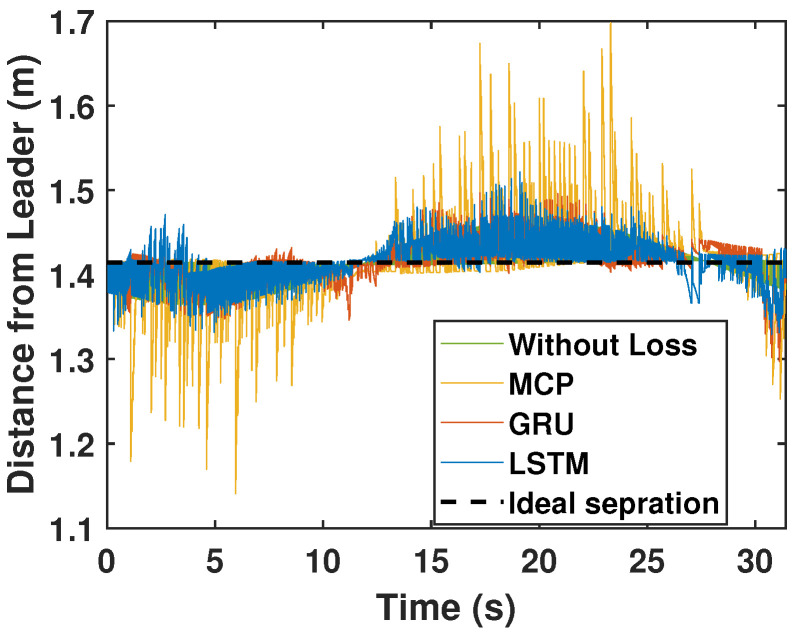
Distance of the follower from the leader for the circular trajectory (packet loss—50%).

We repeated the circular trajectory scenario with a 50% packet loss. The follower performance is illustrated in Figure 10. LSTM again outperformed GRU and MCP in terms of prediction. The distance from the leader is depicted in Figure 11 and the MCP’s performance is compared to those of LSTM and GRU. RMSE was calculated for the X and Y positions and the heading angle of the follower in Table 3 for a 50% packet loss. Here, LSTM outperformed GRU and MCP; while packet loss was 50%, LSTM performance was only slightly worse than it was with 0% loss. Overall, LSTM performed 21.41% better than GRU and 223.17% better than MCP.

#### 6.2.2. Eight-Shaped Trajectory

**Figure 12:**
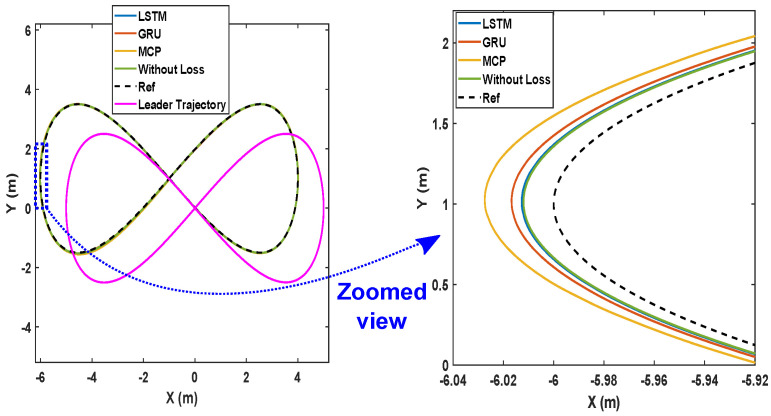
Trajectory of the robots during formation control (packet loss—30%).

**Figure 13:**
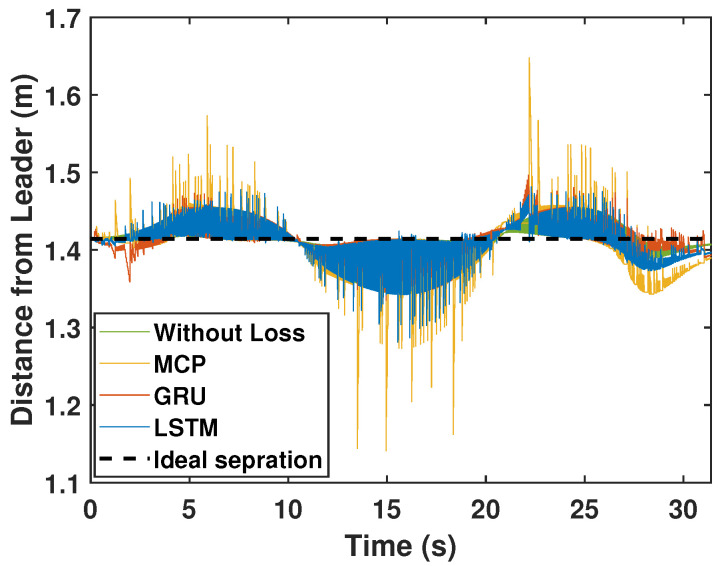
Distance of the follower from the leader for the circular trajectory (packet loss—30%).

Here, we detail the formation control performance of the leader and the followers while following an eight-shaped trajectory in different packet loss scenarios. Figure 12 shows the system’s performance while the packet loss was 30%. It is clearly visible that the LSTM prediction was very close to the perfect communication scenarios when compared with GRU and MCP. The distance from the leader is depicted in Figure 13. Table 4 gives the RMSE for the X, Y, and heading angle of the followers. LSTM performance (RMSE of X, Y, and heading angle) while having 30% packet loss was even better than it was in the perfect communication scenarios (0% packet loss). This demonstrates that LSTM prediction can completely compensate for packet loss and even compensate for the quantization error in the leader positions due to discrete communication intervals. Overall, LSTM performed 5.20% better than GRU and 156.14% better than MCP.

**Figure 14:**
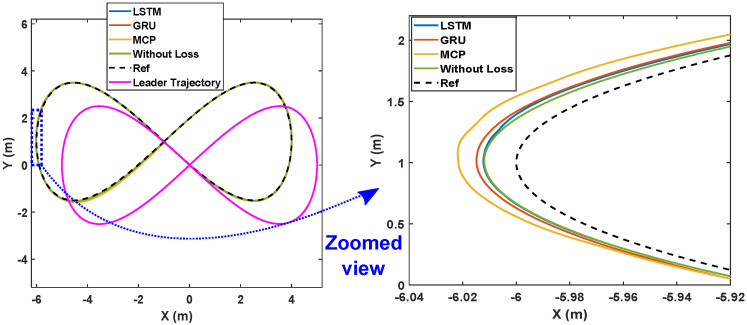
Trajectory of the robots during formation control (packet loss—50%).

**Figure 15:**
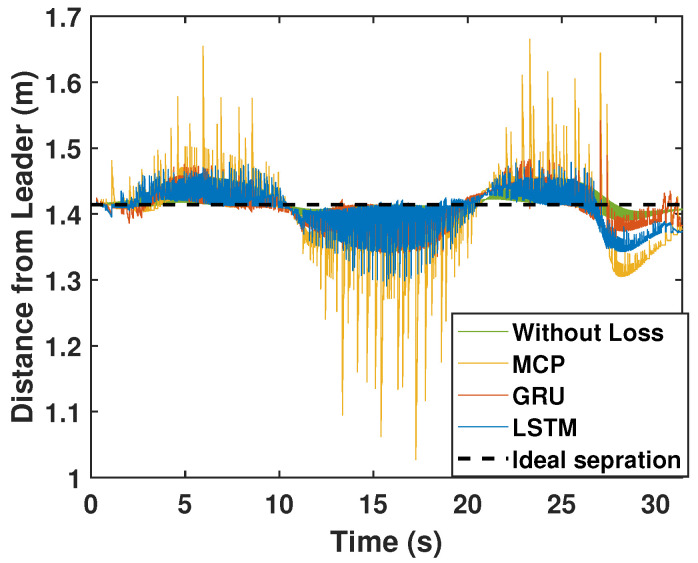
Distance of the follower from the leader for the circular trajectory (packet loss—50%).

The formation control experiment along the eight-shaped trajectory was repeated with 50% packet loss. As illustrated in Figure 14, LSTM again outperformed GRU and MCP in terms of prediction. The distance from the leader is depicted in Figure 15. MCP had a weaker performance compared to LSTM and GRU. The RMSE for the X, Y, and heading angle of the follower is presented in Table 4 for the 50% packet loss scenario. As observed earlier, LSTM outperformed GRU and MCP. LSTM’s prediction performance is comparable with that in a perfect communication scenario (0% packet loss) even when sustaining 50% packet loss. Overall, LSTM performed 14.49% better than GRU and 250.53% better than MCP.

## 7. Conclusions

The formation control problem of non-holonomic AGVs is presented in this study. Decentralized formation control of multiple AGVs is presented based on leader-follower formation control by an LQR controller. The performance of the LQR controller is analyzed when sustaining packet loss and with packet loss compensation using an LSTM neural network model. The LSTM algorithm is in charge of forecasting the leader’s position based on the previous leader’s position. When packet loss occurs, followers rely on LSTM-generated predicted position values to maintain their formation accurately. Numerous simulations were run to compare the performance of the LSTM to that of MCP and GRU. LSTM prediction significantly aids in compensating for packet loss along a variety of trajectories. Overall, LSTM performs 12% better than GRU and 194% better than MCP. In future research, we will consider communication delay and other details of connectivity aspects and plan to also implement our proposed approach in a physical robot test environment.

## Figures and Tables

**Table 1:** RMSE of prediction error.

Packet Loss		30%			50%	
**RMSE**	**X**	**Y**	**Heading Angle**	**X**	**Y**	**Heading Angle**
LSTM	3.19 × 10${}^{-2}$	3.46 × 10${}^{-2}$	9.8 × 10${}^{-3}$	3 × 10${}^{-2}$	3.71 × 10${}^{-2}$	1.01 × 10${}^{-2}$
GRU	3.35 × 10${}^{-2}$	3.52 × 10${}^{-2}$	1.17 × 10${}^{-2}$	3.41 × 10${}^{-2}$	3.72 × 10${}^{-2}$	1.43 × 10${}^{-2}$
MCP	3.47 × 10${}^{-2}$	4.28 × 10${}^{-2}$	1.10 × 10${}^{-2}$	5.53 × 10${}^{-2}$	5.70 × 10${}^{-2}$	1.59 × 10${}^{-2}$

**Table 2:** LSTM and GRU model setting.

LSTM	X	Y	Heading Angle
Epox	400	400	400
Hidden nodes	10	10	7
Batch size	128	128	128
Learning rate	0.005	0.01	0.1
Learning rate drop factor	0.2	0.2	0.2

**Table 3:** RMSE of follower error for the circle path.

Packet Loss	Prediction Method	RMSE-X	RMSE-Y	RMSE-Heading Angle
0%	-	2.76 × 10${}^{-2}$	2.77 × 10${}^{-2}$	7.8 × 10${}^{-3}$
	LSTM	2.45 × 10${}^{-2}$	2.81 × 10${}^{-2}$	8.3 × 10${}^{-2}$
30%	GRU	2.55 × 10${}^{-2}$	3.07 × 10${}^{-2}$	9.7 × 10${}^{-3}$
	MCP	6.23 × 10${}^{-2}$	7.20 × 10${}^{-2}$	1.94 × 10${}^{-2}$
	LSTM	2.39 × 10${}^{-2}$	3.04 × 10${}^{-2}$	9.1 × 10${}^{-3}$
50%	GRU	2.60 × 10${}^{-2}$	3.49 × 10${}^{-2}$	1.28 × 10${}^{-2}$
	MCP	8.94 × 10${}^{-2}$	9.15 × 10${}^{-2}$	2.68 × 10${}^{-2}$

**Table 4:** RMSE of follower error for the eight-shaped path.

Packet Loss	Prediction Method	RMSE-X	RMSE-Y	RMSE-Heading Angle
0%	-	2.39 × 10${}^{-2}$	2.74 × 10${}^{-2}$	1.25 × 10${}^{-2}$
	LSTM	2.08 × 10${}^{-2}$	2.63 × 10${}^{-2}$	1.18 × 10${}^{-2}$
30%	GRU	2.18 × 10${}^{-2}$	2.58 × 10${}^{-2}$	1.33 × 10${}^{-2}$
	MCP	5.35 × 10${}^{-2}$	6.67 × 10${}^{-2}$	3.04 × 10${}^{-2}$
	LSTM	2.09 × 10${}^{-2}$	2.77 × 10${}^{-2}$	1.45 × 10${}^{-2}$
50%	GRU	2.57 × 10${}^{-2}$	2.67 × 10${}^{-2}$	1.80 × 10${}^{-2}$
	MCP	7.94 × 10${}^{-2}$	9.36 × 10${}^{-2}$	4.84 × 10${}^{-2}$
